# Fatal dual infection with *Salmonella* and *Mycobacterium avium* complex infection in a patient with advanced acquired immunodeficiency syndrome: a case report

**DOI:** 10.4076/1757-1626-2-6773

**Published:** 2009-09-11

**Authors:** Adetunji Adejumo, Olutayo Olabige, Vel Sivapalan

**Affiliations:** 1 Division of Infectious Diseases, Department of Medicine, Harlem Hospital/Columbia University, 506 Lenox Avenue, New York, NY 10037, USA

## Abstract

Non-typhoid *Salmonella* and *Mycobacterium avium* complex infections are part of the constellation of infections seen with increasing frequency in patients with acquired immuned deficiency syndrome. The incidence has reduced significantly since highly active antiretroviral therapy era, but their critical nature is unchanged. The co-existence of these infections and the accompanied increased mortality is presented in this case report.

## Case presentation

A 29-year-old African American woman with AIDS (CD4 count of 8 cells/uL) presented with three weeks history of weakness, non-bloody, non-mucoid diarrhea and esophageal candidiasis. Diarrhea was associated with abdominal cramp but no nausea, vomiting and fever. She also had subjective weight loss, non productive cough and anorexia due to difficulty with swallowing. She had no history of recent travel, dietary change or antibiotic use. AIDS was diagnosed two years prior to admission during a routine screening. She had an unclear HIV treatment at the time and multiple opportunistic infections including *Pneumocystis jiroveci* pneumonia and oropharyngeal candidiasis. She was non adherent to *Mycobacterium avium* and pneumocystis prophylactic treatment.

On examination, she was chronically ill appearing, emaciated but in no acute distress. The blood pressure was 115/59 mmHg, pulse 129 beats per minute, respiration 24 breaths per minute, and temperature 100 F, saturation in room air was 99%.

Pertinent findings were marked pallor, extensive oral thrush and massive hepato-splenomegaly (spleen was 8 cm from subcostal margin and liver was 6 cm). Rectal examination was normal. Blood study showed white count 6,000/uL, neutrophil 92%, lymphocyte 4.5%, hemoglobin 7 g/dL, hematocrit 20%, reticulocyte count was 4.39%, ferritin was 617 ng/ml, transferrin saturation of 2.7%, folate 2.41 ng/ml and platelet count was 209,000/uL.

Serum chemistry was normal. The hepatic panel revealed normal aspartate transaminase 26 U/L, normal alanine transaminase 16 U/L, elevated alkaline phosphatase 662 U/L, total bilirubin of 0.7 mg/dL, direct bilirubin 0.2 mg/dL, protein was 4.2 g/dL and albumin 1.5 g/dL. Hepatitis B and C serology was negative.

Blood culture was positive for *Salmonella* newport. Stool culture also grew *Salmonella species*. Blood mycobacterium culture was pending but blood mycology was negative.

Abdominal computed tomography (CT) scan with contrast showed thickening of the wall of proximal small bowel and ascending colon with massive hepatosplenomegaly.

She was started on intravenous ciprofloxacin which was planned for 6 weeks duration because of severity and persistence of salmonella infection in AIDS patients.

Her hospital course was complicated by severe anemia (hematocrit was 14%) attributed to multifactorial etiology including nutritional, anemia of chronic disease and hypersplenism which required 10 units of packed red blood cells over two weeks. Patient refused bone marrow biopsy. Splenectomy was considered but deferred because she was a high surgical risk.

After three weeks of intravenous ciprofloxacin treatment, blood and stool cultures were negative for salmonella and the patient was discharged on oral ciprofloxacin. Antiretroviral therapy with tenofovir 300 mg/emtricitabine 200 mg and lopinavir 400 mg/ritonavir 100 mg had been initiated as well as atovaquone, azithromycin for pneumocystis and *Mycobacterium avium* prophylaxis respectively.

She was recalled nine days later for positive *Mycobacterium avium* complex in a blood culture that was pending at the time of her discharge. On readmission she reported unresolved diarrhea and abdominal cramp. Her vital sign was blood pressure of 70/50 mm of Hg, heart rate of 109 beat per minute, and temperature of 95.5 0F. She was icteric with marked pallor. Laboratory data showed pancytopenia (hemoglobin 6 g/dl, white cells 2,300/uL, platelet count 16,000/uL) and metabolic acidosis. The serum lactate dehydrogenase was 1542 IU/L and D-Dimer was 3156.47. All cultures were repeated.

**Figure 1:**
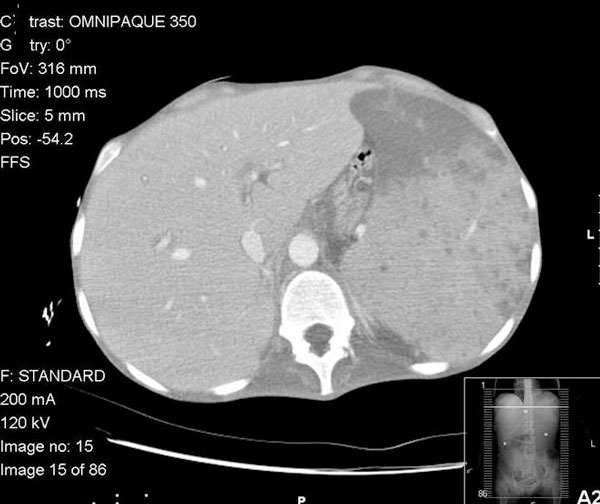
**Abdominal CT scan with intravenous contrast showing hepatomegaly and enlarged spleen with multiple hypo dense lesions**.

Abdominal CT scan with intravenous contrast showed markedly enlarged spleen with multiple hypo dense lesions, moderate ascites, thickened bowel loops, retroperitoneal and periportal lympadenopathy (Figure 1).

She was admitted into the intensive care unit (ICU) for septic shock. Treatment for disseminated MAC was initiated with rifabutin, ethambutol, azithromycin. Intravenous ciprofloxacin was continued for recurrent *salmonella* gastroenteritis. Despite commencing appropriate antimicrobial therapy, she expired on the third ICU day from severe sepsis. Her family refused autopsy.

## Discussion

Concomitant infection with S*almonella* and *Mycobacterium avium* complex as described in this case report is uncommon. Our review of the literature confirmed the rarity of a dual infection at any point in time. The mechanisms that accounted for this dual infection is linked to the uncontrolled HIV viremia with altered cell-mediated immunity. Recurrent s*almonella* infection is an AIDS defining illness that results from incomplete clearance of the primary infection because of impaired cell mediated immunity.

Usual clinical presentation is chronic non bloody diarrhea with associated abdominal cramps. In the United States, between 30 and 60% of HIV-infected patients have diarrhea severe enough to require medical attention at some time during the course of infection [1]. The incidence of chronic diarrhea from all causes in patients with CD4 < 200 cells/uL remain unchanged in the post highly active antiretroviral therapy (HAART) era, ranging from 8 to 10.5% per year [2].

Other important diarrhea causing enteric pathogens in AIDS patients are CMV, *Cryptosporidium*, *Microsporidium*, *Entamoeba histolytica*, *Girdiasis*, and *Clostridium difficile* toxin.

Clinical features of MAC and *Salmonella* enteritis can be very similar and confusing. Both pathogens are known to cause diarrhea disease, low grade fever, hepato-splenomegaly and anemia.

Non-typhoid *Salmonella* (NTS) infections are severe and recurrent in HIV infected adults and are the commonest cause of hospital admission with bacteremia in the Sub Sahara Africa [3]. NTS bacteremia typically presents in patients with AIDS once CD4 count falls below 200 cells/uL. In-patient mortality can be up to 35-60% and is highest in patients with severe anemia and confusion. Other extra-intestinal manifestations of salmonellosis are endocarditis, septic arthritis, pneumonia, abscess, pyomyositis, and pyelonephritis among others.

Among AIDS patient on HAART, the incidence of *Salmonella* and other opportunistic infectious etiology has declined from 53% to 13%, likely as a result of the direct bactericidal activity of the HAART on *Salmonella species* and immune reconstitution [2].

Definitive diagnosis of salmonellosis requires the isolation of the organism from the blood, bone marrow, stool, urine or intestinal secretions. The sensitivity of blood culture is 50-70%; so optimum approach to diagnosis is to culture the blood, bone marrow and intestinal secretions which have 90% yield for diagnosis. In the case described, the patient had positive blood and stool for *Salmonella*. Serology is not useful.

In contrast, disseminated *Mycobacterium avium* complex (MAC) infection is a common complication of advanced AIDS. It is established that MAC is associated with shortened survival in AIDS causing a 3-fold increased risk of death irrespective of CD4 count [4]. The increased mortality may be explained by up-regulation of HIV RNA and/or by direct effects of MAC.

Clinical manifestations include fever, weight loss, night sweat, elevated LDH, elevated alkaline phosphatase and anemia. Factors associated with developing MAC are younger age and antiretroviral therapy naive [5]. Since the advent of HAART, the rate of MAC infection has declined substantially, but patients with low CD4 count remain at risk. Current recommendation requires routine use of chemoprophylaxis with azithromycin 1200 mg weekly in patients with CD < 50 cells/uL and it can be discontinued when CD4 count rises above 100 cells/uL on two consecutive determinations in response to HAART therapy [6]. Life long prophylaxis is recommended for those with history of disseminated MAC without evidence of immune reconstitution or HAART induced rise in CD4 cell count [7].

Diagnosis of MAC is made by isolating the organism from culture of blood, lymph node, liver, spleen and bone marrow (BM). The average duration of culturing depends on the medium used but varies from 7-16 days. AFB staining of BM aspirate is the least sensitive method but results are available in a mean of 1.1 days [8]. In our case culture came back after two weeks.

The standard treatment for disseminated MAC is by combination pharmacotherapy using clarithromycin 500 mg bid, ethambutol 15 mg/kg/d and rifabutin 300 mg/d given indefinitely or until evidence of immune reconstitution secondary to HAART. The United States Public Health Service (USPHS) and Infectious Disease Society of America (IDSA) guidelines state that at least 12 months of therapy and six months of immune reconstitution. Azithromycin 600 mg daily can be used in place of clarithromycin, but clarithromycin is the preferred agent. Other treatment regimen exists.

To avoid treatment delay and poor outcome, clinicians should begin empirical anti-MAC therapy once disseminated MAC is suspected. It should be remembered that a rapid diagnosis using the BM aspirate AFB staining is possible if patient consents.

In conclusion, the occurrence of multiple opportunistic infections should be suspected in late stage AIDS because dual infections will likely increase mortality. These patients are at risk for multiple infections and therefore other causes of a clinical syndrome should be sought even if one cause has been identified, especially if the patient does not improve. AIDS patients must be managed for opportunistic infections and then initiate HAART when feasible as severe immune reconstitution may also be life threatening.

## Abbreviations

AFB: acid fast bacilli; AIDS: acquired immunodeficiency syndrome; BM: bone marrow; CT: computed tomography; HAART: highly active antiretroviral therapy; ICU: intensive care unit; IDSA: infectious disease society of America; MAC: *Mycobacterium avium* complex; NTS: non-typhoid salmonella; USPHS: United States public health service.

## Consent

Written informed consent was obtained from the patient for publication of this case report and accompanying images. A copy of the written consent is available for review by the Editor-in-Chief of this journal.

## Competing interests

The authors declare that they have no competing interests.

## Authors' contribution

AA has a major role in writing manuscript and analyzing laboratory data. OO has a role in literature review and manuscript writing. VS supervised and edited manuscript throughout. All authors read and approved the final manuscript.
